# Efficacy of Wearable Single-Lead ECG Monitoring during Exercise Stress Testing: A Comparative Study

**DOI:** 10.3390/s24196394

**Published:** 2024-10-02

**Authors:** Hyo-In Choi, Seung Jae Lee, Jong Doo Choi, GyungChul Kim, Young-Shin Lee, Jong-Young Lee

**Affiliations:** 1 Division of Cardiology, Department of Internal Medicine, Sungkyunkwan University School of Medicine, Kangbuk Samsung Hospital, Seoul 03181, Republic of Korea; drhyoin.choi@samsung.com (H.-I.C.); sj0519.lee@samsung.com (S.J.L.); 2 Seers Technology Co., Ltd., Seongnam-si 13558, Republic of Korea; andy.choi@seerstech.com (J.D.C.); eric.kim@seerstech.com (G.K.); luke.lee@seerstech.com (Y.-S.L.)

**Keywords:** electrocardiography, wearable technology, exercise test, biomedical technology

## Abstract

Background and Objectives: Few comparative studies have evaluated wearable single-lead electrocardiogram (ECG) devices and standard multi-lead ECG devices during exercise testing. This study aimed to validate the accuracy of a wearable single-lead ECG monitor for recording heart rate (HR) metrics during graded exercise tests (GXTs). Methods: A cohort of 50 patients at a tertiary hospital underwent GXT while simultaneously being equipped with wearable single- and conventional multi-lead ECGs. The concordance between these modalities was quantified using the intraclass correlation coefficient and Bland–Altman plot analysis. Results: The minimum and average HR readings between the devices were generally consistent. Parameters such as ventricular ectopic beats and supraventricular ectopic beats showed strong agreement. However, the agreement for the Total QRS and Maximum RR was not sufficient. HR measurements across different stages of the exercise test showed sufficient agreement. Although not statistically significant, the standard multi-lead ECG devices exhibited higher noise levels compared to the wearable single-lead ECG devices. Conclusions: Wearable single-lead ECG devices can reliably monitor HR and detect abnormal beats across a spectrum of exercise intensities, offering a viable alternative to traditional multi-lead systems.

## 1. Introduction

Cardiovascular diseases (CVDs) pose a substantial global health burden, highlighting the need for advanced and comprehensive management strategies [1]. Among these management strategies, cardiac rehabilitation (CR) is pivotal in facilitating the adoption of an active lifestyle and mitigating cardiovascular risk, thereby enhancing patient outcomes [2]. Central to the success of CR is exercise training, which induces favorable physiological adaptations and promotes cardiovascular health [3]. Therefore, the Frequency, Intensity, Time, and Type (FITT) principles are crucial in physicians’ recommendations for exercise sessions, wherein precise heart rate (HR) monitoring is critical in optimizing exercise efficacy [4]. Accurate HR monitoring is crucial, because even a 10–20 bpm error can significantly impact both the effectiveness and, more importantly, the safety of exercise rehabilitation. For example, in a patient recovering from a myocardial infarction, the initial target heart rate (HR) is often set at 80–96 bpm. If the HR increases by just 10–20 bpm beyond this range, it could result in a sudden increase of over 10–20% in exercise intensity, potentially raising the risk of complications such as recurrent myocardial infarction or arrhythmias. This precision is essential for ensuring optimal patient outcomes.

Multi-lead electrocardiograms (ECGs) have traditionally been considered the gold standard for HR monitoring during clinical exercise stress testing [5]. However, the practical accessibility of ECGs in real-world settings during exercise remains limited [6]. As such, alternative HR monitoring methods have emerged, including handheld [7], chest strap [8], and wearable technologies [9]. Among these, wearable single-lead ECG devices have emerged as promising candidates for ambulatory HR monitoring, allowing for convenient, continuous cardiac data collection over extended periods [10]. Recent reports have highlighted the efficacy and accuracy of wearable single-lead ECGs, with patients expressing a preference for these devices over standard multi-lead ECG monitoring owing to their heightened usability and compliance with cardiac recordings [10]. Furthermore, wearable single-lead ECGs exhibit excellent concordance with ECG parameters and cardiologist arrhythmia diagnoses, bolstering their potential as viable alternatives for HR monitoring during exercise rehabilitation [10].

We aim to validate the accuracy of the wearable single-lead ECG device in clinical settings, particularly during exercise stress testing for patients, where more rigorous validation is necessary compared to its general use in sports activities.

## 2. Methods

This prospective, single-center, consecutive cohort study was conducted at Kangbuk Samsung Hospital, Seoul, Republic of Korea. This study included a consecutive cohort of 50 patients who underwent diagnostic GXTs between September 2021 and December 2021. The patient population was diverse and included individuals with suspected coronary artery disease (CAD), post-confirmation CAD follow-up, suspected arrhythmias, heart failure evaluations, and exercise capacity assessments. The patients were meticulously screened to ensure they could safely complete the treadmill exercise test. Individuals with pacemakers, implantable cardioverter defibrillators, cardiac resynchronization therapy devices, or other medical conditions that could affect the study outcomes were excluded. Informed consent was obtained from all participants before their involvement, and the study protocol was approved by the Institutional Review Board of Kangbuk Samsung Hospital (KBSMC IRB 2020-08-002-002).

**Figure 1:**
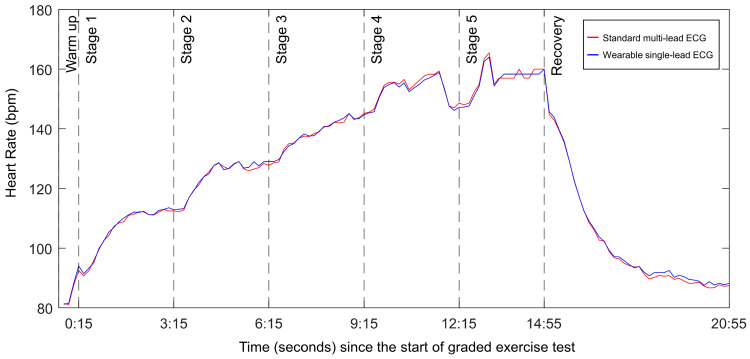
Example of RR intervals for one participant throughout the test. The dotted lines represent different test segments.

The GXT was performed on a T-2100 treadmill (GE Marquette Medical Systems, Milwaukee, MI, USA) following the modified Bruce protocol. This protocol involved adjustments in the treadmill’s incline and speed at three-minute intervals. The test comprised distinct phases: rest, exercise, and recovery. Figure 1 shows the temporal HR profile of a representative participant during the GXT period. For comprehensive cardiac monitoring, 12-lead ECG data were acquired using a CASE Cardiosoft V6.61 system (GE Healthcare). HRs were recorded at 20-s intervals throughout the exercise until the completion of the test. Termination of the test was determined based on either the patient’s request owing to subjective symptoms (such as severe dyspnea, dizziness, leg fatigue, and chest pain) or the attainment of specific physiological endpoints. These endpoints included maximal HR, exercise-induced hypotension, significant hypertension (systolic blood pressure (BP) > 250 mmHg or diastolic BP > 120 mmHg), or abnormal ECG findings, such as pronounced ST-segment depression, increased ventricular ectopy, emergent high-grade atrioventricular block, sustained ventricular tachycardia, or ventricular fibrillation, following the guidelines of the American Heart Association [11].

### 2.1. Wearable Single-Lead ECG Device

The participants were instructed on the use of mobiCARE-MC100, an adhesive single-lead ECG monitoring device manufactured by Seers Technology, Seongnam-si, Gyeonggi-do, Republic of Korea. The MC-100 was equipped with 2 adhesive electrodes spaced 120 mm apart, enabling the recording of single-lead ECGs at a sampling rate of 256 Hz. This device operates on a replaceable battery with a minimum lifespan of 72 h and can transmit ECG data to a preinstalled smartphone application in real time through Bluetooth connectivity. In this study, the MC-100 was strategically positioned 45° from the internal line on the precordium to optimally record lead II signals. This placement adhered to the recommended guidelines for the optimal positioning of wearable single-lead ECG devices [12]. Supplementary Table S1 details the specifications of the MC-100, including its design, operational features, and data transmission capabilities. In addition to evaluating the HR measurement capability of the wearable single-lead ECG, we also assessed its precision and reliability by analyzing ventricular ectopic beats (VEBs), supraventricular ectopic beats (SVEBs), and noise ratio. R-peak detection was performed automatically using a proprietary software algorithm implemented in Python (version 3.10.14; Python Software Foundation, https://www.python.org, accessed on 1 December 2023), and the results were manually cross-validated by experts.

### 2.2. Statistical Analysis

The signal features, including noise, VEBs, and SVEBs, were identified using a dedicated signal comparison tool, enabling expert clinicians to interpret and distinguish each ECG signal. Since the two devices in the study had different sampling rates (200 Hz for the standard multi-lead ECG and 256 Hz for the wearable single-lead ECG), the sampling rate of the standard multi-lead ECG data was adjusted to 256 Hz before interpretation to ensure consistency in the analysis. The noise ratio is defined as the percentage of the total measurement time that corresponds to the duration of sections identified as noise, calculated as ((Noise Section Time/Total Measurement Time) × 100). HR for both the 12-lead ECG and the single-lead ECG was calculated as an average over 20 s, ensuring that the comparison between the two methods was based on the same time frame. In this study, data were expressed as mean ± standard deviation (SD), median with interquartile range, or frequencies and percentages (*n*, %), depending on the context. The agreement between the two ECG devices for individual parameters was assessed using intraclass correlation coefficients (ICCs) and 95% confidence intervals (CIs), with an ICC value > 0.9 indicating high agreement. A comparative analysis of the variables between the devices was conducted using either a paired *t*-test or a Wilcoxon signed-rank test based on the normality of the data distribution. Furthermore, a comprehensive Bland–Altman analysis was conducted using the ‘BlandAltmanLeh’ R package [13]. The analysis involved graphically representing the differences between the measurements from the two devices relative to their mean values. The Limits of Agreement (LoA) were computed, showing that 95% of the differences fell within this range [14]. Paired *t*-tests were used to compare the mean HR values between the wearable single-lead ECG and the conventional 12-lead ECG at each stage of exercise intensity. Paired *t*-tests were conducted using the 20-s mean HR values to precisely capture fluctuations at each stage of exercise intensity.

## 3. Results

### 3.1. Baseline Characteristics

Table 1 shows the baseline demographic and clinical characteristics of the study population (*n* = 50). The average age was 57.8 years, with males constituting 64.0% of the cohort. In terms of comorbidities, 22.0%, 26.0%, 30.0%, 44.0%, and 54.0% of the participants were diagnosed with atrial fibrillation, heart failure, diabetes mellitus, hypertension, and CAD, respectively. The primary indications for undergoing GXT included suspected CAD in 18.0% of patients, confirmed CAD in 50.0%, suspected arrhythmia in 8.0%, evaluation of exercise capacity in 12.0%, and heart failure assessment in 12.0%. Regarding medication use, 36.0%, 28.0%, and 14.0% of the patients were on beta blockers, calcium channel blockers, and antiarrhythmics, respectively. Exercise test results showed that the average maximal metabolic equivalents (METs) achieved were 9.1, with a mean exercise duration of 6.5 min and a maximal HR of 149.5 beats per min (bpm).

The values are expressed as mean ± standard deviation or number (%). BMI indicates body mass index; AMI indicates acute myocardial infarction; DM indicates diabetes mellitus; PCI indicates percutaneous coronary intervention; CABG indicates coronary artery bypass graft; GXT indicates graded exercise test; VO2AT indicates oxygen consumption at anaerobic threshold; VO2max indicates maximal oxygen consumption; METsmax indicates maximal metabolic equivalents; RPEmax indicates maximal rate of perceived exertion; RER indicates respiratory exchange ratio.

### 3.2. Comparison of ECG Monitoring Methods

Of the 50 patients initially included in this study, 3 were excluded from the comparative analysis because of poor ECG acquisition quality. This resulted in a final sample size of 47 patients for analysis. The two ECG monitoring methods—the standard multi-lead and wearable single-lead ECG monitoring devices—are compared in Table 2.

The data are *n* (%), mean ± standard deviation, or median (interquartile range).

The abbreviations include the following: ECG—electrocardiogram; VEB—ventricular ectopic beat; SVEB—supraventricular ectopic beat.

**Figure 2:**
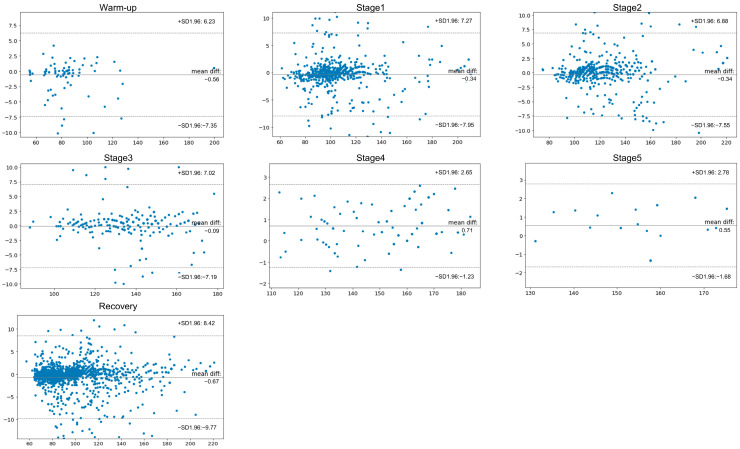
Bland–Altman plots for heart rate during the exercise stage. These plots were constructed with 95% confidence intervals (CIs) for the limits of agreement and mean differences. In this analysis, a wearable single-lead ECG device was used as a reference standard. The *X* axis represents the means, and the *Y* axis represents the difference. Abbreviations: CI—confidence interval; ECG—electrocardiogram; HR—heart rate; ICC—intraclass correlation coefficient; SD—standard deviation.

The analysis revealed an exceptional agreement between the two methods in measuring the total count of QRS complexes, VEBs, and SVEBs, as well as their respective burdens. These findings are supported by the high ICC coefficients, indicating the strong reliability of the measurements obtained using both devices. Moreover, there was a notable level of agreement for the minimum, average, and maximum HRs, as well as the maximum RR intervals between the two monitoring methods. Supplementary Table S2 presents the mean differences and LoAs for each measured variable. Most variables demonstrated sufficiently small LoAs, around ±10, indicating high agreement, while only the Total QRS and Maximum RR exhibited LoAs exceeding ±50, suggesting greater variability. The larger LoAs for these parameters may be due to increased measurement errors caused by noise in the standard multi-lead ECG devices as the physical activity levels increased. However, non-significant nominal discrepancy was observed in the noise ratio. The noise ratio in the standard multi-lead ECG recordings was approximately 1.3%, whereas it was notably lower at 0.6% in the wearable single-lead ECG device, as shown in Table 2 and the representative image in Figure 2. Although this difference was not statistically significant, a wearable single-lead ECG may be less susceptible to noise interference.

### 3.3. HR Agreement across Exercise Test Stages

**Figure 3:**
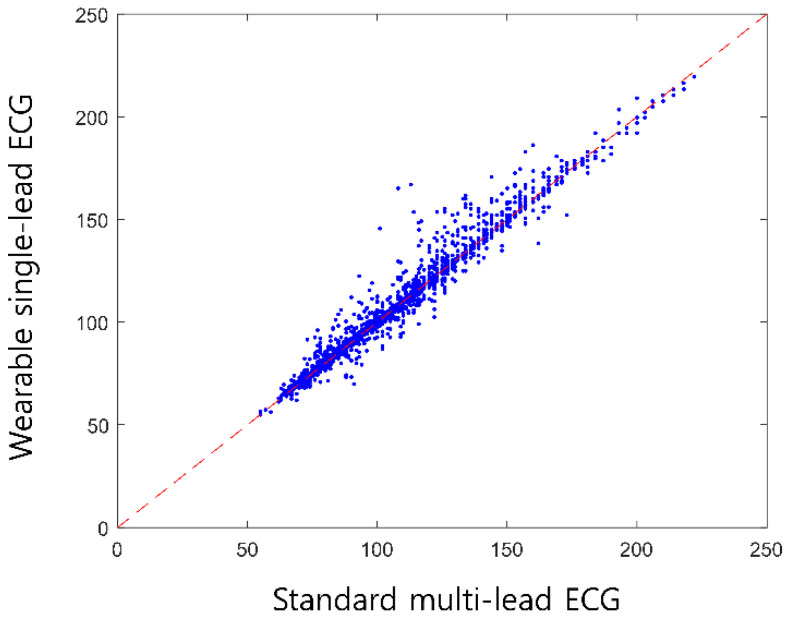
Regression plots depicting the concordance in heart rate (HR) measurements between wearable single-lead and standard multi-lead ECGs. These plots demonstrate a notable agreement between the wearable single-lead and standard multi-lead ECGs in HR monitoring. Abbreviations: ECG—electrocardiogram; HR—heart rate; ICC— intraclass correlation coefficient.

**Figure 4:**
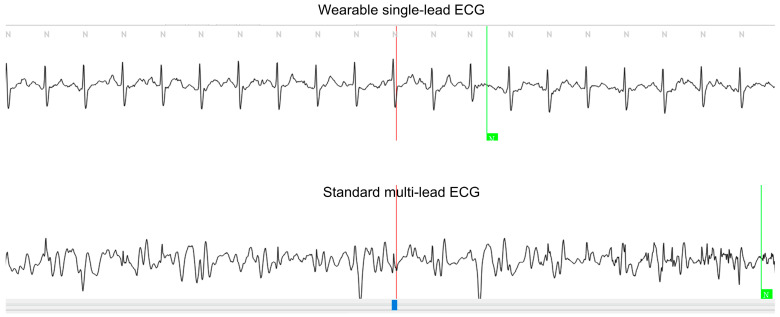
Example of ECG waveforms captured using both wearable single-lead and standard multi-lead ECGs. The upper panel displays the ECG waveform from the wearable single-lead ECG, while the lower panel shows the waveform from lead II of the standard multi-lead ECG. This figure illustrates a representative example where noise was detected in the multi-lead ECG but not in the single-lead ECG, emphasizing the difference in noise sections between the two devices.

Table 3 presents the HR measurements recorded using both the traditional GE and wearable single-lead ECG devices at various stages of the exercise test. The relationship between the traditional multi-lead and wearable single-lead ECGs in terms of HR measurements during each exercise stage is graphically represented in Figure 3. The data showed significant agreement in HR measurements between the two devices throughout the different stages of the exercise test. During the exercise test, the HR increased consistently across all stages, as recorded by both ECG monitoring systems. The highest mean HR was recorded during the final stage, with the standard multi-lead and wearable single-led ECGs reporting peak HRs of approximately 156 and 155 bpm, respectively. Throughout the test, from the warm-up phase to the most strenuous stage, a remarkable level of agreement in HR measurements existed between the two devices. Even as the HR increased to its maximum, the concordance in readings between traditional multi-lead ECG monitoring and the wearable single-lead ECG monitoring devices remained consistently high (Figure 4), indicating their reliability in tracking dynamic cardiac responses during varying levels of physical exertion. The regression plots illustrate a robust correlation between the HR values recorded by both devices, with concordance correlation coefficients approaching 0.99. Supplementary Figure S1 illustrates the distribution of the RR intervals measured during the exercise in a representative case. The left and right panels show the total and fastest RR intervals, respectively. Visually, discernible concordance exists in the measurement of RR intervals between the wearable single-lead and standard multi-lead ECGs.

## 4. Discussion

In this study, wearable single-lead ECG and standard multi-lead ECG monitoring during exercise stress tests were compared. Wearable single-lead ECG and standard multi-lead ECG monitoring during exercise stress tests were compared. Bland–Altman analysis showed that the single-lead ECG had a good correlation with the multi-lead ECG in HR measurements during the tests. Notably, the accuracy of the wearable single-lead ECG was not compromised even at higher exercise intensities, indicating its reliability for cardiac monitoring during increased physical exertion. Additionally, the wearable device demonstrated a tendency for better noise section detection, likely owing to reduced motion artifacts, thus highlighting its potential advantage over standard multi-lead ECG monitoring in providing accurate HR measurements during physical activity. The HR is fundamental in guiding cardiovascular exercise and maintaining overall cardiovascular health, as it allows for precise regulation of exercise intensity and effective monitoring of heart health. The Bland–Altman analysis yielded LoAs that were below ±10 bpm across all stages, which is sufficient for general exercise intensity monitoring. However, in clinical settings such as CR, especially for patients with myocardial infarction, even small discrepancies in HR accuracy could impact both safety and exercise efficacy. Thus, while our results indicate good overall agreement, these LoA results should be cautiously considered for precise rehabilitation programs.

CR is an essential component of CVD management and should be individualized according to the needs of each patient. It is particularly recommended for patients with ischemic heart disease, heart failure, and myocardial infarction or for those who have undergone interventions such as coronary angioplasty or coronary artery bypass grafting [15]. Recent clinical guidelines strongly support exercise-based CR given significant benefits in reducing mortality and morbidity associated with CVD [11]. Despite these benefits, referral to and participation in supervised center-based exercise-based CR programs remain suboptimal [16]. Home-based CR programs have been introduced to overcome barriers to traditional CR participation and offer viable alternatives [17]. These programs have demonstrated effects comparable to center-based CR in reducing mortality, minimizing the risk of recurrent coronary events, and managing cardiovascular risk factors [18,19]. However, home-based CR programs have limitations, including the absence of exercise supervision and the challenge of providing optimized, individualized exercise prescriptions that can adapt to daily changes in a patient’s condition. Additionally, significant concerns remain regarding the safety of home-based CR programs, particularly for patients requiring close monitoring [20]. Wearable single-lead ECG devices for CR monitoring offer significant benefits [21]. Accurate exercise parameter measurements from these devices can assist in tailoring personalized exercise regimens and optimizing the intervention intensity for individual patients [22]. This is especially beneficial for individuals with low exercise confidence or who are apprehensive about resuming exercise after a cardiac event. This study primarily focused on the use of wearable single-lead ECGs in clinical exercise stress testing, where expert oversight is available to verify signal quality. Further research is needed to assess the utility and accuracy of wearable single-lead ECG devices in out-of-hospital settings, where such oversight may not be readily available.

The development of various wearable HR-monitoring devices has significantly advanced exercise training [23]. Previous studies have assessed the accuracy of different monitoring systems, including chest strap monitors [24] and adhesive patch-type single-lead ECG monitors [25]. Chest strap monitors, for instance, align with ECG readings during specific exercises such as treadmill use or stationary cycling [24] but may be less comfortable for extended use. Alternative HR-monitoring technologies include smart bands and smartwatches that utilize photoplethysmography (PPG) [26]. PPG devices measure blood volume changes by using a photodetector placed against the skin [26]. However, these devices are prone to artifacts due to their inherent detection mechanisms, and accuracy decreases with increasing exercise intensity [27]. Meanwhile, electrode-based chest monitors generally provide more precise readings [7]. Single-lead ECG monitoring devices offer a more comfortable and less intrusive alternative for improving patient adherence and comfort during exercise. In this study, a wearable single-lead ECG device certified by the Korea Ministry of Food and Drug Safety demonstrated comparable accuracy to standard multi-lead ECG systems. Furthermore, the wearable single-lead ECG provided reliable HR measurements during exercise stress tests that were comparable to those obtained with traditional ECG methods. This concordance was sustained, even at higher exercise intensities. Notably, the standard multi-lead ECG monitor with its longer wires exhibited a larger noise section than a wearable single-lead ECG device. While standard multi-lead ECGs are essential for detecting parameters such as ST-level deviations in myocardial infarction patients during exercise stress tests, this study focused on the accuracy of HR monitoring. Future research should investigate the efficacy of single-lead ECG devices in detecting more complex markers like ST deviations, which require multiple leads for precise interpretation.

## 5. Limitations

This study had several limitations. First, it was conducted at a single institution with a relatively small cohort of patients. This limitation may hinder the generalizability of our findings to broader populations and more diverse healthcare contexts. Additionally, the reliance of this study on simulated exercise routines compared to monitoring actual physical activities in daily life scenarios may not completely capture the real-world performance of wearable single-lead HR monitoring devices. Consequently, further research involving larger and more diverse groups of participants is required. Studies conducted in real-world exercise environments may provide more definitive insights into the practical applicability and broader relevance of these devices for exercise training.

In this consecutive prospective cohort study involving patients with CVD, the wearable single-lead ECG demonstrated good concordance with the standard multi-lead ECG in terms of HR measurement and abnormal beat detection. These findings suggest that wearable single-lead ECGs can aid exercise prescription.

## Figures and Tables

**Table 1:** Baseline characteristics of the study population (*n* = 50).

	Value
Demographics	
Age, year	57.8 ± 11.2
Male, %	32 (64.0)
Height, cm	164.3 ± 10.0
Weight, kg	66.2 ± 15.6
Body mass index, kg/m^2^	24.3 ± 4.2
Comorbidity	
Hypertension	22 (44.0)
Diabetes mellitus	15 (30.0)
Coronary artery disease	27 (54.0)
Atrial fibrillation	11 (22.0)
Heart failure	13 (26.0)
Peripheral artery disease	1 (2.0)
Chronic kidney disease	3 (6.0)
Chronic obstructive pulmonary disease	1 (2.0)
Cerebrovascular accident	0 (0.0)
Indication for exercise test	
Suspected coronary artery disease	9 (18.0)
Known patients with coronary artery disease	25 (50.0)
Suspected arrhythmia	4 (8.0)
Evaluation of exercise capacity	6 (12.0)
Evaluation of heart failure	6 (12.0)
Medications	
Beta blocker	18 (36.0)
Calcium channel blocker	14 (28.0)
Antiarrhythmic drug	7 (14.0)
Exercise test results	
Maximal METs	9.1 ± 3.3
Exercise duration, mins	6.5 ± 3.1
Maximal heart rate, bpm	149.5 ± 27.2

**Table 2:** Comparisons of ECG monitoring between 12-lead ECG and adhesive single-lead ECG monitoring for the study population.

	12-Lead ECG	Wearable Single-Lead ECG	*p*-Value for Reliability (Correlation)	Corrcoef	*p*-Value for Difference
Total QRS	1405.9 ± 540.7	1419.3 ± 542.5	<0.001	>0.9	0.19
VEB	15.2 ± 66.1	15.4 ± 66.1	<0.001	1.0	0.0078
VEB burden, %	0.01 ± 0.06	0.01 ± 0.06	<0.001	1.0	>0.9
SVEB	3.6 ± 8.6	3.6 ± 8.6	<0.001	1.0	>0.9
SVEB burden, %	0.004 ± 0.009	0.004 ± 0.009	<0.001	1.0	>0.9
Max RR, ms	931 ± 250	931 ± 245	<0.001	>0.9	>0.9
Average RR, ms	570 ± 93	569 ± 92	<0.001	>0.9	0.34
Min RR, ms	357 ± 73	357 ± 73	<0.001	1.0	>0.9
Noise ratio, %	1.3 ± 3.7	0.6 ± 2.1	<0.001	0.41	0.13

**Table 3:** HR means of wearable single-lead ECG device compared to conventional 12-lead ECG by exercise intensity.

Stage	Conventional 12-Lead ECG		Wearable Single-Lead ECG		N	ICC		*p*-Value for Difference
	Mean in HR (bpm)	SD	Mean in HR (bpm)	SD	N	r	*p*-Value	*p*-Value
Warm-up	85	23.7	86	23.5	90	0.990	<0.001	0.45
Stage1	107	24.0	107	24.3	826	0.987	<0.001	0.29
Stage2	124	23.0	124	23.2	645	0.987	<0.001	0.31
Stage3	137	17.1	137	17.2	335	0.976	<0.001	0.25
Stage4	153	17.4	153	17.2	123	0.998	<0.001	0.45
Stage5	156	12.1	155	11.9	26	0.995	<0.001	0.45
Recovery	101	27.9	101	28.4	1695	0.986	<0.001	0.37

Abbreviations: HR—heart rate; ECG—electrocardiogram; SD—standard deviation; ICC—intraclass correlation coefficient.

## Data Availability

Data are contained within the article.

## Supplementary Materials

The following supporting information can be downloaded at: https://www.mdpi.com/article/10.3390/s24196394/s1. Supplementary Table S1: The specification of the MC-100. Supplementary Table S2: Mean Differences and Limits of Agreement for Each Measured Variable. Supplementary Figure S1: Example of ECG monitoring by wearable single-lead ECG and conventional multi-lead ECG for one participant. (a) ECG tracing recorded by a wearable single-lead ECG device, illustrating clear QRS complex recognition; (b) ECG tracing recorded by a conventional multi-lead ECG system, showing poorly distinguished QRS complexes, primarily represented as noise.
