# Supplementary material for: Efficacy of Wearable Single-Lead ECG Monitoring during Exercise Stress Testing: A Comparative Study
Source: Sensors (Basel). 2024 Oct 2;24(19):6394. doi: 10.3390/s24196394 (PMC11479017; doi:10.3390/s24196394)
Supplement: Supplementary file 1 [file sensors-24-06394-s001.zip › sensors-3180628-supplementary.pdf]

Supplementary Table S1. The specification of the MC-100.

Manufacturer	Seers Technology (Seongnam-si, Gyeonggi-do, Republic of Korea)
Product serial number	mobiCARE-MC100
Product appearance	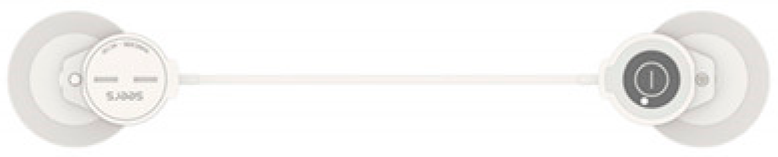
Size	Width 29 mm, length 120 mm
Weight	8.9 g
Measurements	Single-lead electrocardiogram in real-time, heart rate, movement activity
Sensors	Electrodes, accelerometers, gyroscopes
Connectivity	Bluetooth low energy
Heart rate measurement range	From 30 to 240 beat-per-minutes
Sampling rate	256 Hz
Battery	Replaceable CR2032H coin cell battery
Electrode standard	Medical standard 4.0 mm electrode snaps for electrocardiogram
Operating time	Lasting at least 72 h continuously

Supplementary Table S2. Mean Differences and Limits of Agreement for Each Measured Variable

	Mean difference	+ SD1.96	- SD1.96	LOA
Total QRS	13.38	140.98	-114.21	255.19
VEB	0.17	0.91	-0.57	1.48
VEB burden	0	0	0	0
SVEB	0	0	0	0
SVEB burden	0	0	0	0
Noise ratio	-0.74	5.6	7.08	-1.48
Minimum RR	0	0	0	0
Average RR	-0.94	11	-13	24
Maximum RR	0.13	65	-65	130
Warm-up	-0.56	6.23	-7.35	13.58
Stage 1	-0.34	7.27	-7.95	15.22
Stage 2	-0.34	6.88	-7.55	14.43
Stage 3	-0.09	7.02	-7.19	14.21
Stage 4	0.71	2.65	-1.23	3.88
Stage 5	0.55	2.78	-1.68	4.46
Recovery	-0.67	8.42	-9.77	18.19

* LoA, limits of agreement; VEB, ventricular ectopic beats; SVEB, supraventricular ectopic beats; bpm, beats per minute.
