# Supplementary figures and images for: Efficacy of Wearable Single-Lead ECG Monitoring during Exercise Stress Testing: A Comparative Study
Source: Sensors (Basel). 2024 Oct 2;24(19):6394. doi: 10.3390/s24196394 (PMC11479017; doi:10.3390/s24196394)

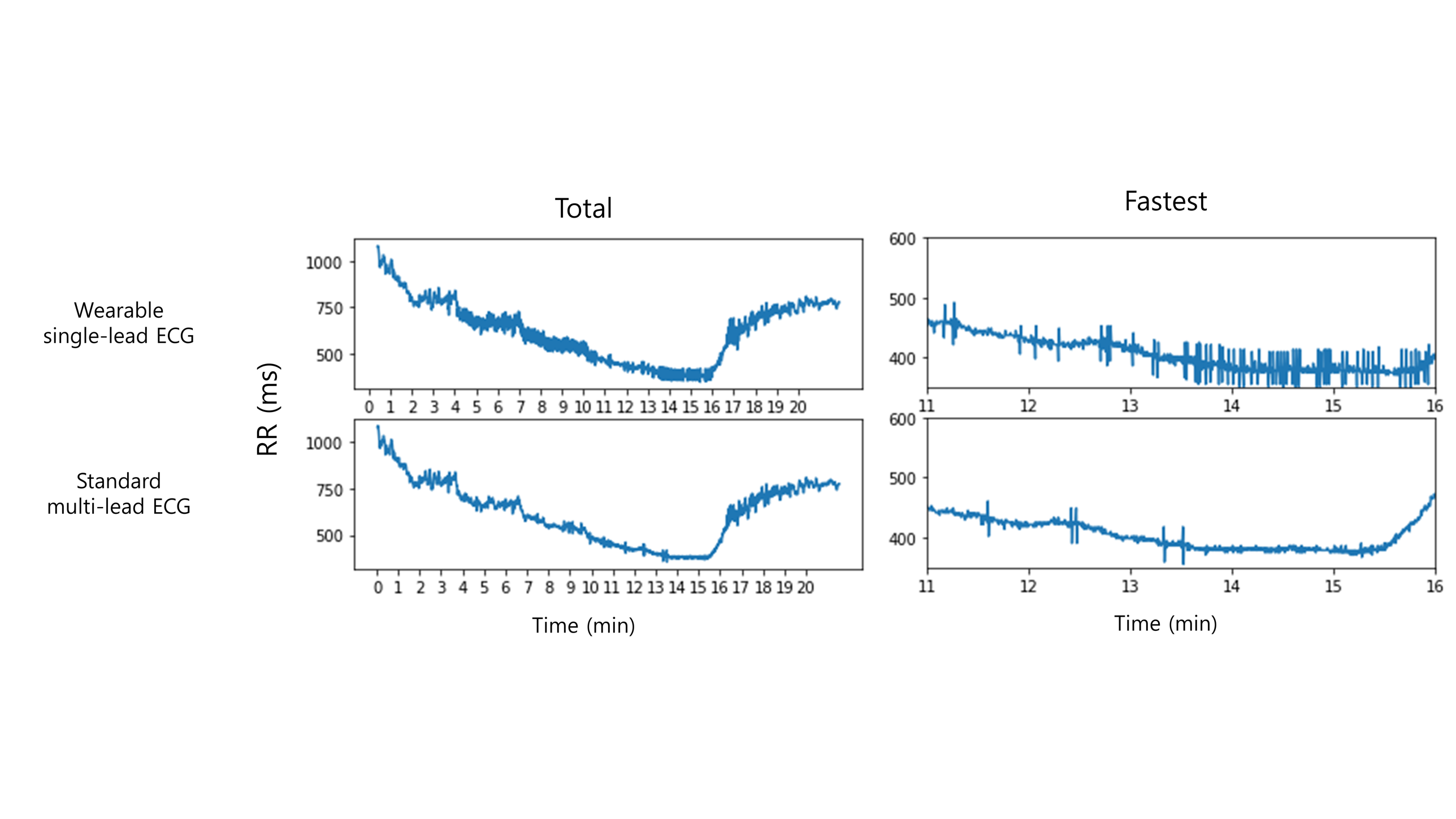

Supplement: Supplementary file 1 [file sensors-24-06394-s001.zip › Supplementary figure.tif]
